# DeepLRR: An Online Webserver for Leucine-Rich-Repeat Containing Protein Characterization Based on Deep Learning

**DOI:** 10.3390/plants11010136

**Published:** 2022-01-04

**Authors:** Zhenya Liu, Zirui Ren, Lunyi Yan, Feng Li

**Affiliations:** 1 Key Lab of Horticultural Plant Biology (MOE), College of Horticulture and Forestry Sciences, Huazhong Agricultural University, Wuhan 430070, China; 2 College of Informatics, Huazhong Agricultural University, Wuhan 430070, China; alexrenzr@gmail.com (Z.R.); lunyiyan@foxmail.com (L.Y.)

**Keywords:** deep learning, LRR domain, plant disease-resistance genes

## Abstract

Members of the leucine-rich repeat (LRR) superfamily play critical roles in multiple biological processes. As the LRR unit sequence is highly variable, accurately predicting the number and location of LRR units in proteins is a highly challenging task in the field of bioinformatics. Existing methods still need to be improved, especially when it comes to similarity-based methods. We introduce our DeepLRR method based on a convolutional neural network (CNN) model and LRR features to predict the number and location of LRR units in proteins. We compared DeepLRR with six existing methods using a dataset containing 572 LRR proteins and it outperformed all of them when it comes to overall F1 score. In addition, DeepLRR has integrated identifying plant disease-resistance proteins (NLR, LRR-RLK, LRR-RLP) and non-canonical domains. With DeepLRR, 223, 191 and 183 LRR-RLK genes in Arabidopsis (Arabidopsis thaliana), rice (Oryza sativa ssp. Japonica) and tomato (Solanum lycopersicum) genomes were re-annotated, respectively. Chromosome mapping and gene cluster analysis revealed that 24.2% (54/223), 29.8% (57/191) and 16.9% (31/183) of LRR-RLK genes formed gene cluster structures in Arabidopsis, rice and tomato, respectively. Finally, we explored the evolutionary relationship and domain composition of LRR-RLK genes in each plant and distributions of known receptor and co-receptor pairs. This provides a new perspective for the identification of potential receptors and co-receptors.

## 1. Introduction

The plant immune system is mainly composed of two mechanisms [1]. The first deploys a large number of receptor-like kinases (RLKs) and receptor-like proteins (RLPs) as plasma membrane pattern recognition receptors (PRRs) that detect microbe-associated molecular patterns (MAMPs). This mechanism is named PAMP-triggered immunity or PTI [2]. Among them, the leucine-rich repeats containing RLKs (LRR-RLKs) and leucine-rich repeats containing RLPs (LRR-RLPs) play crucial roles in plant growth, development, signal transduction, immunity and stress response [3,4]. Plant LRR-RLKs are plasma membrane proteins composed of an extracellular domain (LRR domain), a single-pass transmembrane domain and a cytoplasmic kinase domain. On the other hand, a LRR-RLP is essentially a LRR-RLK lacking a cytoplasmic kinase domain [5]. The other mechanism relies on the specific recognition and interaction behavior between plant disease resistance (R) proteins and pathogen effectors. This mechanism is called effector-triggered immunity (ETI) [6]. The nucleotide binding leucine-rich repeat proteins (NLRs) encoded by plant R genes can recognize the pathogen effector, thereby triggering a plant immune response [7]. Plant NLRs usually contain a C-terminal LRR domain and a central NB-ARC domain (nucleotide-binding adaptor shared by Apaf-1, resistance proteins and CED-4) [8]. In addition, plant NLRs are roughly divided into two groups, depending on whether their *N*-terminal contains a Toll/interleukin-1 receptor (TIR) domain, TNLs or non-TNLs. Some non-TNLs have a coiled coil domain composed of CNLs [9,10]. Proteins with two or more LRR units form a LRR superfamily, including intracellular, extracellular and membrane-bound proteins with diverse functions involving multiple biological processes [11].

Plants use LRR-RLKs residing on the cell surface to sense different external and internal cues and elicit distinct biological responses. The signaling pathway mediated by LRR-RLKs enlists a small group of co-receptors, such as somatic embryogenesis receptor kinases (SERKs), through ligand-induced heterodimerization and transphosphorylation. In Arabidopsis, receptor and co-receptor pairs mainly regulate signal transduction in plant cell differentiation, growth and immunity. Brassinosteroid insensitive 1 (BRI1) interacts with SERK1, SERK3/BAK1 and SERK4/BKK1 to sense plant hormone brassinosteroids (BRs) for regulation of plant growth [12,13,14,15]. LRR-RLK PSKR1 interacts with SERK3/BAK1 and PSK-induced root growth as well as cell expansion are inhibited in SERK3/BAK1 mutants [16,17]. The secreted EPF peptides interact with LRR-RLK ER and its homologue ER-LIKE1 (ERL1) to negatively regulate the stomatal pattern [18]. The study showed all four functional SERKs were necessary, but their genetic contribution to stomatal patterns decreased from SERK3, SERK2, SERK1 and finally to SERK4 [19]. Floral organ abscission is regulated by the secreted peptides IDA, LRR-RLK HAE, HAE-LIKE 2 (HSL2) and the downstream MAP kinase (MAPK) cascade signaling pathway in Arabidopsis [20]. Genetic analysis of SERK mutants showed that SERKs redundantly regulated HAE and HSL2-mediated floral organ abscission. SERK1, SERK2, SERK3 and SERK1, SERK3 and SERK4 mutants exhibit floral organ shedding defects [21,22]. SERK1 and SERK2 redundantly regulate male gametophyte development [23,24]. It has been proposed that the LRR-RLK excess microsporocytes 1 (EMS1)/extra sporogenous cells (EXS) form a complex with SERK1 and SERK2. This senses the secreted peptide tapetum determinant 1 (TPD1) to regulate male gametophyte development [25]. Among well-characterized MAMPs, a conserved 22-aa peptide (flg22) and an 18-aa peptide (elf18) are perceived by LRR-RLK FLS2 and EFR, respectively [26,27]. SERK3/BAK1 interacts with FLS2 or EFR to sense flg22 or elf18 [28,29,30]. Genetic data revealed that SERK3/BAK1 is the main player, while SERK4 only works in SERK3/BAK1 mutants. The role of SERK1 and SERK2 is negligible in plant immunity, although all four functional SERKs form complexes with FLS2 and EFR [31]. Endogenous plant elicitor peptides (Peps) serve as damage-associated molecular patterns (DAMPs) to enhance defense responses [32]. LRR-RLK PEP1 receptors 1 and 2 (PEPR1 & PEPR2) interact with SERK3/BAK1 to sense AtPep1 in a manner similar to FLS2 and EFR. SERK3/BAK1 and SERK4 redundantly regulate the AtPep1 signal pathway because ethylene accumulation and ROS production triggered by AtPep1 are eliminated in the SERK3/BAK1 double mutants, but not in the single mutant [33,34,35].

LRR is a conserved domain with characteristic hydrophobic leucine residues present in the majority of immune receptors such as NLR, RLK and RLP [36]. Each LRR unit usually consists of 20 to 30 amino acids (aa) divided into a highly conserved segment (HCS) and a highly variable segment (HVS). The HCS usually contains a characteristic eleven aa pattern “LxxLxLxxNxL” where x represents any aa. Other hydrophobic residues replace L with certain probability at each conserved position [37].

Due to their high level of sequence variability necessitated by the diverse functions of LRR domains, it is a challenging task in bioinformatics research field to predict the exact position and number of LRR units in a protein. Current LRR prediction tools can be divided into two categories: sequence similarity-based and de novo prediction tools. Sequence similarity-based tools usually build the HMM model or PSSM matrix via sequence alignment, such as Pfam [38], SMART [39], Prosite [40], LRRfinder [41] and LRR search [42]. De novo prediction tools like LRRpredictor [43] are based on supervised learning models (e.g., SVM, MLP and AdaBoost). De novo prediction methods based on traditional machine learning algorithms usually have low prediction power. This is because their ability to extract hidden sequence characteristics is weak and the training dataset provides a limited representation of actual data.

Recently, the application of deep learning algorithm in bioinformatics increased exponentially [44] and its great potential was demonstrated in many research fields. These include prediction of gene expression [45], prediction of binding sites in RNA binding proteins [46] and interaction sites between proteins [47]. Due to the powerful capability of deep learning and importance of LRR repeats in biological activities, we developed DeepLRR. This is a deep learning-based web server that can predict potential LRR domains and units in protein sequences as well as characterize plant NLR, RLK and RLP resistance proteins and their non-canonical domains. Using DeepLRR, this study re-annotated LRR-RLK genes in Arabidopsis, rice and tomato. It further explored the distribution of known receptor and co-receptor pairs on the phylogenetic tree and provides a new perspective for identification of potential receptors and co-receptors.

## 2. Materials and Methods

### 2.1. DeepLRR Overview

DeepLRR predicts potential LRR domains and units in a protein sequence through concerted local and global analyses. In the local analysis phase, LRR protein sequences are retrieved from the Swiss-Prot database and annotated LRR unit sequences in these proteins are collected to form the positive sample dataset. To construct the negative sample dataset, we predict the highly conserved segment pattern (HSCP) of LRR units on the basis of the constructed positive sample dataset. The negative sample dataset was then artificially generated by eliminating HSCP. Thereafter, we use positive and negative sample datasets to build training, validation and testing datasets to train and test our CNN model. This is then used to evaluate any peptide sequences between 20 to 30 aa in length and to give a LRR unit probability score (LPS) for each peptide sequence. A given protein sequence is divided into 11 groups of short sequences based on a 20 to 30 aa-long sliding window and each short sequence is evaluated based on whether its LPS is a LRR unit. In the global analysis, we combined the CNN model and LRR features to set the dynamically adjustable parameters Lscp, Ldcp and Lncp, respectively. These parameters help us determine whether the multiple LRR units predicted by the CNN model can form a LRR domain. In addition, we have integrated the identification process of NLR, RLK and RLP resistance proteins and their non-canonical domains to improve the applicability of DeepLRR.

### 2.2. LRR Positive Sample Dataset Construction

To ensure the reliability of the LRR positive sample used in this study, 1849 protein sequences containing 18,039 LRR units are downloaded from the Swiss-Prot database.

As most LRR units are 20 to 30 aa in length, we filtered LRR units with a length less than 20 aa or a length greater than 30 aa and retain protein sequences with at least three LRR units. To date, we have obtained a total of 1748 protein sequences containing 17,388 LRR units. The last step is to use CD-HIT [48] to remove redundancies from 17,388 LRR units with 80% as the threshold. After the above steps, we obtain a total of 10,938 LRR units as the LRR positive sample dataset (Supplementary Figure S1).

### 2.3. LRR Negative Sample Dataset Construction

Due to the particularity for the negative LRR unit samples, they cannot be downloaded directly from the existing database and so we artificially create the dataset. The first step is to use MEME-5.1.0 [49] to predict the highly conserved segment pattern (HCSP) in 10,938 LRR units. Following this, MAST is used [50] to predict highly conserved segments (HCS) in 10,938 LRR units based on the predicted HCSP. It is worth mentioning that because we know each LRR unit is composed of a HCS and a highly variable segment (HVS), the e-value of MAST is thus set to 1e3 to predict as many HCS as possible in LRR units. In the third step, we performed further statistical analysis on the frequency of different amino acids in each conserved position (aa residue No.1, aa residue No.4, aa residue No.6, aa residue No.9 and aa residue No.11) of the predicted HCSs. Then, we selected the amino acids with a frequency ≥0.5% of appearing in each conserved position to construct a complete HSCP. The fourth step is to download 174,800 protein sequences annotated as not containing LRR units from the Swiss-Prot database. We then randomly cut a certain number of sequence segments from 20 to 30 aa in length and ensure that these segments do not contain the HCSP. CD-HIT was also used to remove redundancy for these non-LRR units and to set the sequence identity threshold at 80%. Finally, the same number of non-LRR units as the LRR units of different lengths in the LRR positive sample dataset are randomly selected as the LRR negative sample dataset (Supplementary Figure S2A).

### 2.4. Training, Validation and Testing Dataset

In order to ensure that the performance of the model is stable and reliable, we build training, validation and testing datasets to train and test the performance of our models. The training dataset is used to train the model, and the validation dataset is used to adjust the parameters and avoid overfitting. After a reasonable number of iterative learning cycles, we test the final performance of the model on an independent test dataset. It is important to note that the testing dataset has never been used in the model during training and validation. The ratio of training dataset to testing dataset values is 8:2. Five-fold cross-validation is performed on the training dataset to test the generalization ability of the model, adjust parameters and avoid overfitting (Supplementary Figure S2B). The proportion of positive and negative samples of different lengths in the training, validation and testing dataset are all 1:1.

### 2.5. Input Matrix

This study does not apply the one-hot encoding commonly used in encoding sequence. Instead, a new sequence encoding method proposed recently is used, and any protein sequence can be encoded as a 20 × 5 binary matrix [51]. For each amino acid there is a corresponding 1 × 5 binary vector (from [0,0,0,0,1] for A to [1,0,1,0,0] for Y). The LRR units involved in this study are all short sequences with a length of no more than 30 aa. If a LRR unit has a length of less than 30 aa, the free amino acid positions will be filled with a binary vector [1,0,1,0,1]. In addition, [1,0,1,1,1] is used to represent amino acids that are not among the 20 common amino acids (O, U, B, Z, J, X).

### 2.6. CNN Model Structure

**Figure 1:**
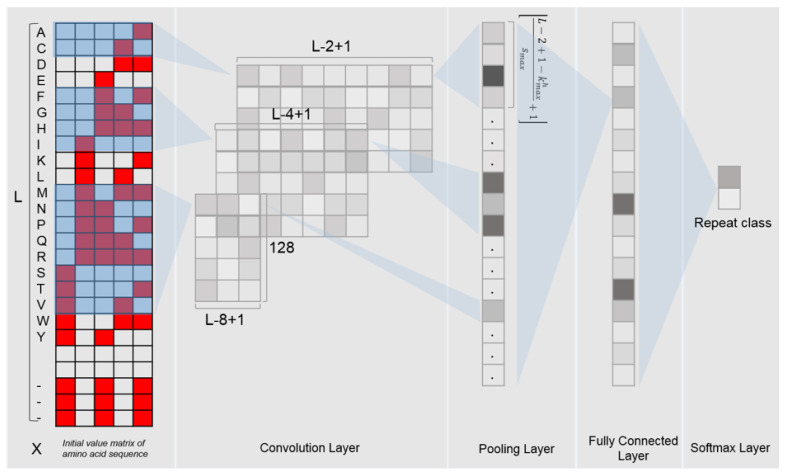
Framework of the DeepLRR CNN model.

The deep learning model used in this study is the convolutional neural network (CNN). The CNN model is implemented with the Python programming language and the Pytorch library, which consists of multiple layers: one convolutional layer, one max pooling layer, one fully connected layer and one softmax layer (Figure 1). In order to fully extract the features of the LRR units, we use 3 different dimensions of convolution filters (for each dimension, there were 128 filters with a step size of 1) to scan the input binary matrix. After the max pooling layer, a vector containing 128*38 outputs for each LRR unit was obtained.

### 2.7. Model Training

During model training, the binary cross-entropy loss function (BCELoss) was minimized for the true label. The negative classes and adaptive momentum (Adam) optimizer (learning rate = 0.0001, β_1_ = 0.9, β_2_ = 0.999, ε = 1 × 10^−8^) is chosen for optimization. The dropout method is used in the fully connected layer and the dropout rate is set to 0.5 to prevent the neural network from overfitting. In addition, we also use the early stop strategy to further prevent the neural network from overfitting. We use 50 epochs with a mini-batch size of 128.

### 2.8. Performance Evaluation

In order to comprehensively assess LRR unit prediction performance, four commonly applied statistical measures are adopted in this work, including sensitivity (Sen), precision (Pre), F1 score and Matthew’s correlation coefficient (MCC). They are defined as follows:

(1) $$\mathrm{Sen}=\frac{TP}{TP+FN}\;$$

(2) $$\mathrm{Pre}=\frac{TP}{TP+FP}\;$$

(3) $$F1\;\mathrm{score}=2\times \frac{Pre\times Sen}{Pre+Sen}\;$$

(4) $$\mathrm{MCC}=\frac{TP\times TN-FP\times FN}{\sqrt{\left(TP+FP\right)\times \left(TP+FN\right)\times \left(TN+FP\right)\times \left(TN+FN\right)}}\;$$

where *TP* represents the number of LRR units correctly classified, *TN* represents the number of non-LRR units correctly classified, *FN* represents the number of LRR units incorrectly classified as non-LRR units and *FP* represents the number of non-LRR units incorrectly classified as LRR units. Sen (also called true positive rate) measures the percentages of LRR units correctly classified; Pre indicates the ratio of true LRR units that are classified as LRR units by DeepLRR; the F1 score comprehensively considers precision and sensitivity; MCC represents the balance quality of the positive and negative data.

### 2.9. LRR Domain Prediction

In the global analysis, potential LRR units (up to 11) at each position along the protein sequence are further assessed to determine which are able to form a LRR domain. To this end, a LRR score control parameter (Lscp) is implemented for quality control. For example, when the CNN model predicts k LRR units starting from the i-th aa of various lengths, these LRR units are kept if k is greater than or equal to Lscp. Otherwise, they are removed. Next, the protein sequences are divided into consecutive 20-aa segments. In each of these 20-aa regions, the LPS of 20-aa LRR units for each position are compared and only the LRR units at the position where the LPS of each 20-aa LRR unit is the largest are kept. A LRR distance control parameter (Ldcp) is then implemented to divide these 20-aa LRR units kept from the previous step into different groups. For example, two adjacent LRR units are considered to be in different groups if the number of aa between them is greater than Ldcp, otherwise they are considered to be in the same group. In the final step, a LRR number control parameter (Lncp) is implemented to filter unqualified LRR groups. For example, if the number of LRR units in a group is greater than or equal to Lncp, this group of LRR units will be considered a potential LRR motif by DeepLRR. The LRR unit with the largest LPS at each position will be returned without overlapping with adjacent LRR units.

### 2.10. Plant Disease Resistance Proteins and Non-Canonical Domains

In this research, we built an integrated pipeline to characterize NLR, RLK and RLP proteins and their associated non-canonical domains based on their domain architecture. The TIR (PF01582), and NB-ARC (PF00931) domains in NLR and kinase domain (PF00069, PF07714) in RLK are identified using Pfam. Transmembrane domains and signal peptides in RLK and RLP are identified using TMHMM [52] and SignalP [53], respectively. The LRR domain in NLR, RLK and RLP is characterized by DeepLRR. Finally, Pfam was used to identify non-canonical domains in NLR, RLK and RLP. It is worth noting that our approach to annotate NLRs using Pfam has a limitation. The CNL cannot be detected in our pipeline because the CC domain is not defined in the Pfam database. To overcome this limitation, we recommend using the COILS [54] webserver to further predict which of the NLRs predicted by our pipeline contains a CC domain.

### 2.11. Re-Annotation of LRR-RLK Genes in Arabidopsis, Rice and Tomato Genomes Based on DeepLRR

To re-annotate the LRR-RLK genes of Arabidopsis, rice and tomato, the identification pipeline of DeepLRR was used to browse the proteome of Arabidopsis (TAIR10.1) [55], rice (IRGSP-1.0) [56] and tomato (ITAG4.1) [57], respectively. In addition, LRR-RLK genes identified in the corresponding plant reference genome and representative paper were collected and compared with our re-annotated results.

### 2.12. Chromosome Mapping, Gene Cluster Analysis and Phylogenetic Analysis

Combined with the gene location information in the reference genome of each plant, 223, 191 and 183 re-annotated LRR-RLK genes were mapped on the chromosomes of Arabidopsis, rice and tomato through Mapchart [58]. Subsequently, gene cluster and tandem repeat analysis were performed on the re-annotated LRR-RLK genes of these three plants. The principle of dividing gene clusters is as follows: (1) The distance between two adjacent LRR-RLK genes is less than 200 kb; (2) The number of non-LRR-RLK genes between two adjacent LRR-RLK genes is no more than 8 [9,59]. The principle of judging tandem repeats is as follows: (1) The distance between adjacent LRR-RLK genes is less than 100 kb; (2) The similarity between adjacent LRR-RLK genes is higher than 70% [60]. The 200–300 aa kinase domain sequence in the re-annotated LRR-RLK gene was used for phylogenetic analysis. These sequences are compiled and aligned using the clustalW algorithm from MEGA-X [61] with default parameters. The phylogenetic trees were generated with the neighbor-joining algorithm [62] using the following parameters: Complete deletion, a Poisson correction model and bootstrap (1000 replicates, random seed). EvolView v3 [63] is used to add annotated datasets for phylogenetic trees.

## 3. Results

### 3.1. Characterization of Highly Conserved Segment Pattern in LRR Units

Although the LRR HCSP has been documented in previous work based on a limited number of LRR proteins, it is necessary to reestablish a new LRR HCSP with the discovery of more and more LRR units. We built an LRR unit HCS pattern “LxxLxLxxNxL” based on the DeepLRR CNN positive sample dataset. In the HCSP, L at the first position can be substituted with hydrophobic aa: I, V, M, T, F; L at the fourth position can be substituted with I, V, F, M; L at the sixth position can be replaced by V, I, F, M, A, C; N at the ninth position can be replaced by C, T, S; L at the 11th position can be replaced by I, F, V, M, S or N with certain probability (Supplementary Table S1). Through this new HSCP, we artificially created the negative sample dataset required for the DeepLRR method.

### 3.2. Comparison of LRR Unit Prediction Performance for Different Models

**Figure 2:**
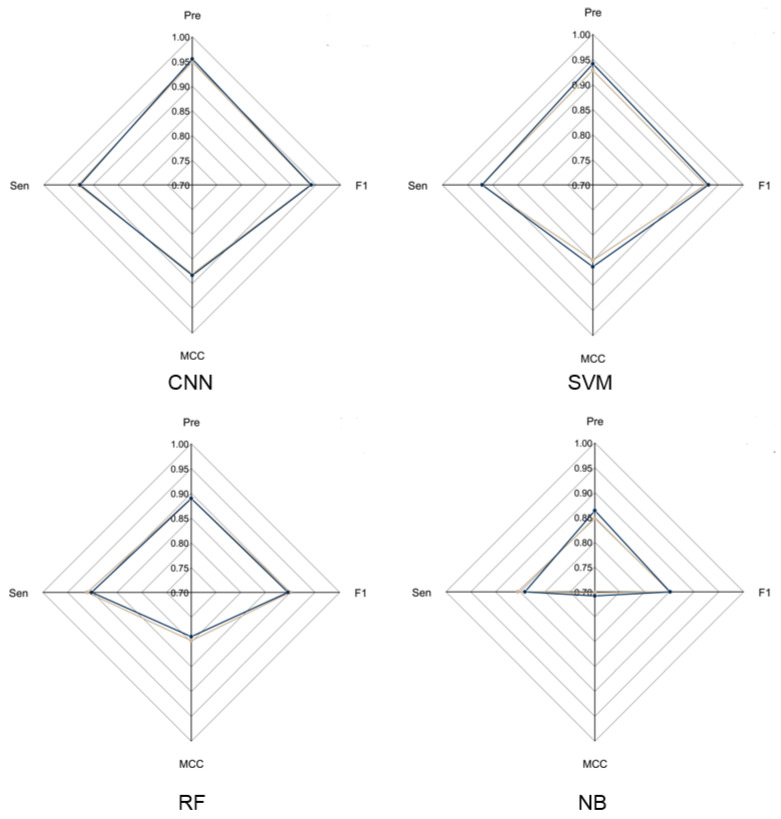
Radar chart of CNN model and three machine learning models. The radar chart shows four evaluation indicators: Precision, Sensitivity, F1-score and MCC. The brown line represents the average performance of the 5-fold cross validation for each model and the dark blue line represents the performance of each model using the test dataset.

To evaluate the overall performance of CNN models in LRR unit prediction, three additional machine learning models were also set up, including supported vector machine (SVM), random forest (RF) and naïve Bayes (NB) models. Five-fold cross validation was conducted to adjust the parameters for these models. The four models were evaluated using an independent testing dataset based on their precision, sensitivity, F1-score and MCC (Figure 2) performance. During this test, the CNN model obtained the highest F1-score of 0.9405, which is 0.01, 0.0452 and 0.0886 higher than that of SVM, RF and NB, respectively. The MCC value of CNN is 0.8831, which is 0.0203, 0.0938 and 0.1751 higher than that of SVM, RF and NB, respectively (Supplementary Table S2). These results showed that the CNN model is more stable and has more robust capability to predict LRR units compared to the other three models.

### 3.3. Optimization of DeepLRR Parameters for LRR Domain Characterization

DeepLRR has three adjustable parameters: Lscp, Ldcp and Lncp. It is essential to understand the impact of these parameters on the performance of DeepLRR. For this, CD-HIT is used to remove the redundancy of 1748 LRR proteins with 80% as the threshold and 1144 non-redundant LRR proteins are divided into two groups. One group was used to evaluate the impact of different combinations for these parameters on DeepLRR performance. The other was used to compare the performance between DeepLRR and six LRR predicting tools currently in use. Based on the overall performance of DeepLRR in the test on the first group of LRR proteins, we found that when parameters Lscp and Ldcp were fixed, the Lncp gradually increased from 2 to 10. The precision in the prediction results of DeepLRR gradually increased and the sensitivity gradually decreased, while the F1-score firstly increased and then decreased. When parameters Lscp and Lncp were fixed, the Ldcp gradually increased from 1 to 20, while the precision in the prediction of DeepLRR firstly increased and then decreased. Sensitivity and F1-score firstly increased and then remained basically unchanged. Finally, when parameters Ldcp and Lncp (Lncp ≤ 4) were fixed, Lscp gradually increased from 1 to 4 and the precision as well as F1-score in the prediction results of DeepLRR gradually increased while the sensitivity was gradually decreased. However, when Lscp gradually increased from 4 to 11, the precision, sensitivity and F1-score in the prediction results of DeepLRR remained basically unchanged. When parameters Ldcp and Lncp (Lncp ≥ 5) were fixed, changing the value of Lscp has almost no effect on the performance of DeepLRR (Supplementary Figure S3). We performed further analysis and found when Lncp was set at 3 or 4, the largest F1-score could be obtained. When Lncp was set at 3 and Lscp [4,11] as well as Ldcp were set [8,13], the F1-scores were all greater than 0.820 with an average value of 0.825 and a maximum value of 0.827 (Lscp = 4, Ldcp = 9–11, Lncp = 3). When Lncp was set at 4, Lscp [4,11] and Ldcp [8,13] were set, the F1-scores were all greater than 0.809 with an average value of 0.817 and a maximum value of 0.828 (Lscp = 4, Ldcp = 11, 12, Lncp = 4). The above results tell us that we should adjust the parameters of DeepLRR depending on research focus. For example, if we pay more attention to the precision of DeepLRR, we need to calibrate the increase in Lscp and Lncp while decreasing Ldcp. Similarly, the trend runs opposite for sensitivity. Some fixed parameter combinations may maximize balance in the prediction performance of the DeepLRR method, such as Lscp/Ldcp/Lncp = 4/9/3 or 4/11/4.

### 3.4. Comparison of DeepLRR Performance with Existing Tools on LRR Domain Characterization

It is necessary to compare the proposed method with other existing state-of-the-art tools. We compared the performance of DeepLRR (Lscp = 4, Ldcp = 9, Lncp = 3) with the six existing tools using the second group of LRR proteins, which have not been used before. Our results showed that the F1-score with DeepLRR is 0.701, 0.241, 0.528, 0.035, 0.024 and 0.071 higher than that with Pfam, Prosite, SMART, LRRfinder, LRRsearch and LRR predictor, respectively. In addition, DeepLRR is the only tool whose precision and sensitivity are both greater than 0.740 (Table 1). This result showed that DeepLRR has better comprehensive performance in LRR unit prediction at protein level compared to the six existing tools.

### 3.5. Webserver Implementation

**Figure 3:**
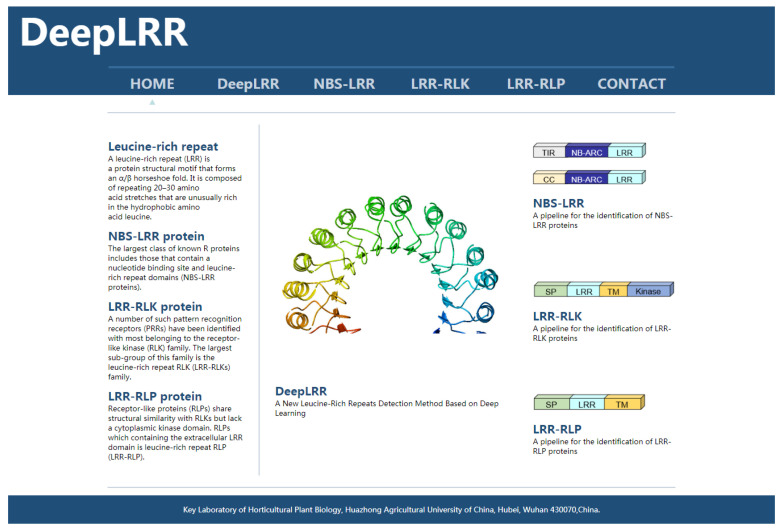
The homepage of the DeepLRR website. The left side of the main body of the website briefly introduces the research focus of DeepLRR while the right side shows the main functional modules of DeepLRR.

To implement the proposed DeepLRR method, we have developed a user-friendly online webserver which is freely available at (http://lifenglab.hzau.edu.cn/DeepLRR/; accessed on 28 November 2021) (Figure 3). If users only need to analyze a single protein sequence, they can paste it in the sequence window in the FASTA format. In order to meet the needs of users, we also provide a multi-sequence analysis button where users can upload their own protein sequence files. The file size is limited to 10 MB. The generated prediction results for all the submitted jobs will be presented in a page and a download link with detailed information about the sequence and the prediction result is provided. In addition, we provide a complete solution for implementing DeepLRR on Linux if users need large-scale and fast analyses.

### 3.6. Re-Annotation of LRR-RLK Genes in Arabidopsis, Rice and Tomato Genomes

**Figure 4:**
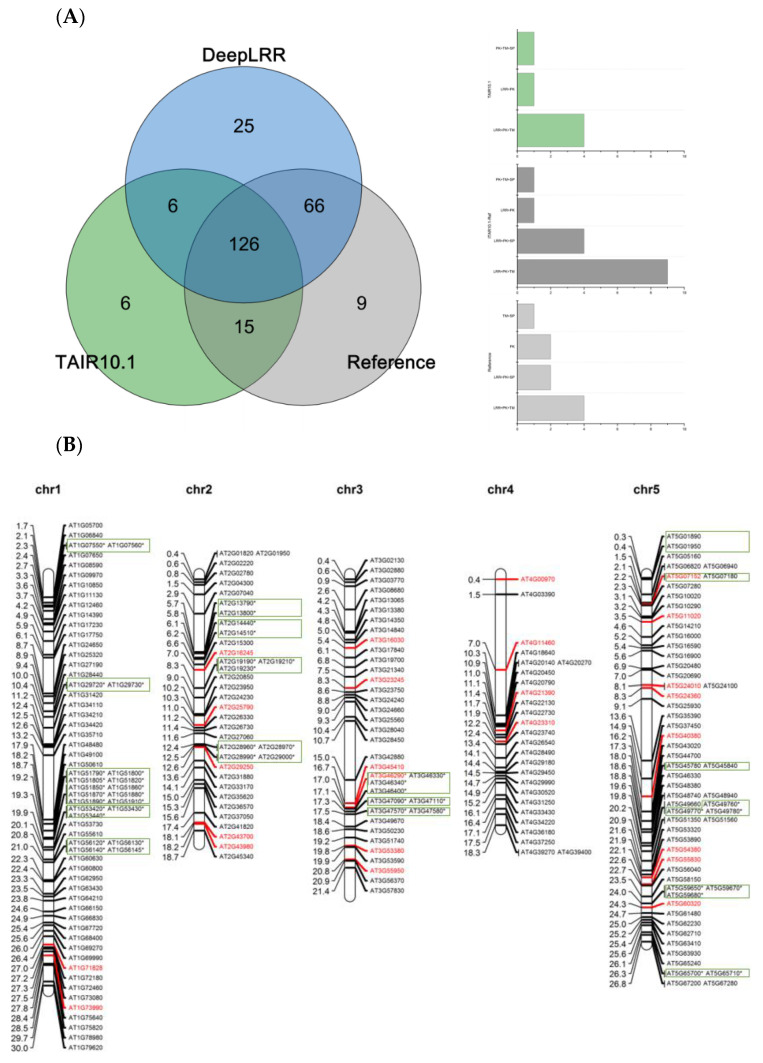

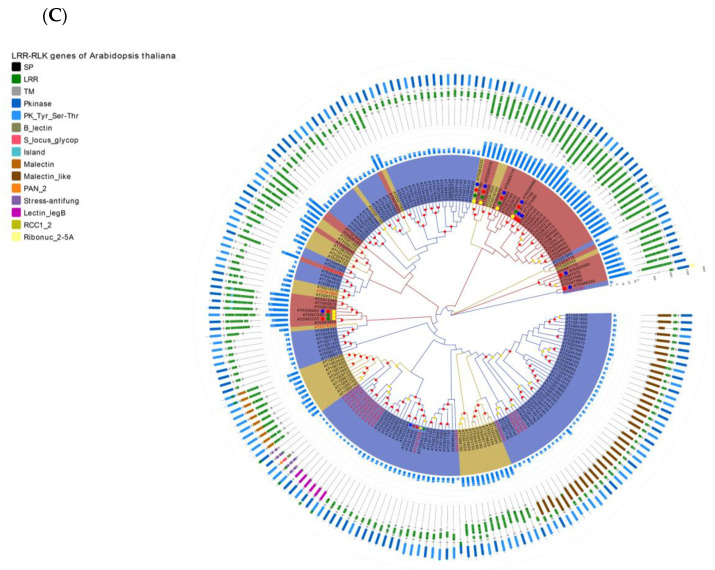
Re-annotation of the LRR-RLK gene in the Arabidopsis genome, chromosome mapping, gene cluster analysis and phylogenetic analysis. (**A**) The Venn diagram on the left shows the annotated results of the LRR-RLK gene in the Arabidopsis genome for DeepLRR, reference genome TAIR10.1 and the representative paper respectively. The histogram on the right shows the domain composition of the LRR-RLK gene that DeepLRR could not successfully annotate, including three datasets. One is unique to TAIR10.1, the other is shared by TAIR10.1 and the representative paper and the last is unique to the representative paper. (**B**) The distribution of LRR-RLK genes was re-annotated by DeepLRR on the chromosomes of Arabidopsis. The green rectangles represent different gene clusters, tandem repeat genes are marked with an asterisk and gene names marked in red are LRR-RLK genes annotated only by DeepLRR. (**C**) An unrooted phylogenetic tree of LRR-RLK genes was re-annotated by DeepLRR in Arabidopsis. The phylogenetic tree was established with amino acid sequences of the kinase domains using the neighbor-joining (NJ) method. The circles with different colors on the sub-nodes of the phylogenetic tree show different ranges of bootstrap values. The red circle shows bootstrap values from 0.9 to 1, the gold circle shows bootstrap values from 0.7 to 0.9 and the dark grey circle shows bootstrap values from 0.5 to 0.7. The different background colors of the leaf nodes indicate that the number of LRR units contained covers different ranges. Dark red indicates that the number of LRR units is greater than or equal to 20, dark yellow indicates that the number of LRR units is greater than or equal to 10 and less than 20, and dark blue indicates that the number of LRR units is less than 10. The histogram outside the leaf node shows the number of corresponding LRR units in detail. In addition, the phylogenetic tree shows the receptor and co-receptor pairs that have been experimentally verified so far. The circle represents a receptor, the triangle represents a co-receptor and the same color indicates that there is an interaction. Finally, the domain composition of each LRR-RLK gene is shown in detail.

Using the LRR-RLK identification pipeline provided by the DeepLRR website service, 223 LRR-RLK genes were identified from Arabidopsis reference genome ITAG10.1. Comparing the annotation results of LRR-RLK genes from DeepLRR, reference genome ITAG10.1 and a representative paper [64], we found that the intersection of these three annotation results contain 126 LRR-RLK genes. DeepLRR and the representative paper have the largest intersection with 192 LRR-RLK genes while only 25 LRR-RLK genes were identified by DeepLRR. We then further analyzed 30 LRR-RLK genes that were not annotated by DeepLRR, with 17 LRR-RLK genes lacking the signal peptide, 6 LRR-RLK genes lacking the transmembrane domain, 5 LRR-RLK genes lacking the LRR and 1 LRR-RLK gene lacking the kinase domain (Figure 4A). Similarly, we re-annotated and analyzed the LRR-RLK genes in rice and tomato reference genomes. DeepLRR identified 191 and 183 LRR-RLK genes in the rice reference genome IRGSP-1.0 and tomato reference genome ITAG4.1 respectively. 14 and 23 LRR-RLK genes have not been included in the reference genome and the representative paper [65,66] respectively. There are 125 LRR-RLK genes in the rice genome that cannot be annotated by DeepLRR. Among them, 34 LRR-RLK genes lack the signal peptide, 8 LRR-RLK genes lack the transmembrane domain, 29 LRR-RLK genes lack the leucine-rich repeat, 43 LRR-RLK gene lack the kinase domain and 10 LRR-RLK genes lack four typical features or domains (Supplementary Figure S4A). It is worth noting that as the rice reference genome version was updated, 40 LRR-RLK genes identified in the representative paper cannot be found in the updated IRGSP-1.0 version. Meanwhile, 114 LRR-RLK genes in the tomato genome cannot be identified using DeepLRR. Among them, 47 LRR-RLK genes lack the signal peptide, 6 LRR-RLK genes lack the transmembrane domain, 11 LRR-RLK genes lack the leucine-rich repeat, 40 LRR-RLK genes lack the kinase domain and 5 LRR-RLK genes lack four typical features or domains at the same time (Supplementary Figure S5A).

### 3.7. Chromosome Mapping, Gene Cluster Analysis and Phylogenetic Analysis

The 223, 191 and 183 LRR-RLK genes identified by DeepLRR were mapped to different chromosomes of Arabidopsis, rice and tomato respectively through Mapchart [58]. The LRR-RLK gene is distributed in different quantities on the chromosomes of Arabidopsis, rice and tomato and is unevenly distributed. Approximately 29.6% (66/223) of LRR-RLK genes in Arabidopsis are distributed on chromosome 1, which is the highest ratio compared with other chromosomes. This is followed by chromosome 5 where there are 58 LRR-RLK genes accounting for about 26% of the total. The chromosome with the least number of genes in Arabidopsis is chromosome 4, which contains 11.7% (26/223) of LRR-RLK genes (Figure 4B). Chromosome 2 in rice contains the largest number of LRR-RLK genes at approximately 13.6% (26/191). Chromosomes 1, 6 and 11 contain the same number of LRR-RLK genes at approximately 12% (23/191). In addition, the number of LRR-RLK genes mapped to rice chromosomes 9, 10 and 12 are all less than 10, which are 3.7% (7/191), 4.7% (9/191) and 3.1% (6/191) respectively (Supplementary Figure S4B). In addition, the number of LRR-RLK genes distributed on the remaining chromosomes is between 10–20. Tomato chromosomes 2 and 3 contain the most number of LRR-RLK genes which is approximately 16.4% (30/183). The number of LRR-RLK genes distributed on chromosomes 5, 10 and 11 are all less than 10, making up 3.8% (7/183), 4.4% (8/183) and 4.4% (8/183) respectively. Similarly, the number of LRR-RLK genes distributed on the remaining chromosomes is between 10–20 (Supplementary Figure S5B).

Genes in the same gene cluster are marked with rectangles and LRR-RLK genes of Arabidopsis, rice and tomato have a clustering characteristic. Eighteen gene clusters were found in Arabidopsis, including 54 LRR-RLK genes, accounting for approximately 24.2% (54/223) of the total. Arabidopsis contains an average of 3.6 gene clusters per chromosome and each gene cluster contains an average of 3 LRR-RLK genes. Among the 5 chromosomes of Arabidopsis, chromosome 5 contains the largest number of gene clusters, with 6 clusters containing 14 LRR-RLK genes in total. The gene cluster on chromosome 1 contains the largest number of LRR-RLK genes with the 5 gene clusters containing a total of 21 LRR-RLK genes. No gene cluster on chromosome 4 was found (Figure 4B). Two of the 25 additional LRR-RLK genes identified by DeepLRR are located in the gene cluster. Twenty-one gene clusters were found in rice, including 57 LRR-RLK genes, which accounted for approximately 29.8% of all relevant genes. Rice contains an average of 1.75 gene clusters per chromosome, and each gene cluster contains an average of 2.7 LRR-RLK genes. Rice chromosome 2 contains the largest number of gene clusters, with 3 gene clusters containing a total of 12 LRR-RLK genes and one gene cluster containing the largest number of LRR-RLK genes, which has 8 LRR-RLK genes. There is no gene cluster on chromosomes 9 and 12. Three of the 14 additional LRR-RLK genes identified by DeepLRR are involved in the establishment of gene clusters (Supplementary Figure S4B). Thirteen gene clusters were found in tomato, including 31 LRR-RLK genes and accounting for approximately 16.9% of the total. Tomato contains an average of 1.08 gene clusters per chromosome and each gene cluster contains an average of 2.4 LRR-RLK genes. Tomato chromosome 2 contains the most gene clusters, with 5 gene clusters containing a total of 12 LRR-RLK genes. In addition, no gene clusters were found on chromosomes 5, 7, 9 and 12. Four of the 23 additional LRR-RLK genes identified by DeepLRR are involved in the gene clusters (Supplementary Figure S5B). It is worth noting that the ratio of LRR-RLK genes for gene clusters in Arabidopsis and rice is 1.43 and 1.76 times that in tomato, respectively.

There are also tandem repeats in the LRR-RLK genes of Arabidopsis, rice and tomato. In Arabidopsis, rice and tomato, we found that 21.5% (48/223), 27.7% (53/191) and 14.2% (26/183) of the LRR-RLK genes were tandem duplications (Figure 4B, Supplementary Figures S4B and S5B). The distance between adjacent LRR-RLK genes in tandem repeats is smaller than that in gene clusters and all tandem repeats appear in gene clusters. In addition, about 88.9% (48/54), 93% (53/57) and 83.9% (26/31) of LRR-RLK genes in Arabidopsis, rice and tomato gene clusters are tandem repeats, which indicates the tandem repeat is the main method by which gene clusters form.

In this work, the LRR-RLK gene with a kinase domain length of 200–300 aa in Arabidopsis, rice and tomato was used for phylogenetic analysis. Phylogenetic analysis found that the number of LRR units for LRR-RLK genes under the same evolutionary branch in Arabidopsis, rice and tomato is mostly distributed the same way (Figure 4C, Supplementary Figures S4C and S5C). It may be that the kinase domain of the LRR-RLK gene and its LRR unit are evolutionarily consistent. It is worth noting that there are many LRR units of individual LRR-RLK genes in the evolutionary branch with less than 10 LRR units. Examples include AT5G10020 and AT2G25790 in Arabidopsis, Os07g0626500 and Os03g0791700 in rice as well as Solyc09g007110 and Solyc02g070000 in tomato. This phenomenon may indicate that these evolutionary branches are evolving in the direction of increasing LRR units. Because LRR-RLK genes containing a large number of LRR units can form a stable bond with MAMPs, it means that these branches will receive more attention from pathogens and will participate more in PTI as PRRs. In the NBS-LRR disease resistance gene, studies have reported that its non-canonical domain is used as a trap domain in the process of recognizing pathogen effectors [67,68]. This research indicates Arabidopsis, rice and tomato LRR-RLK genes all contain six non-canonical domains: B_lectin, S_locus_glycop, Malectin_like, PAN_2, Malectin and Island which account for about 84% (63/75), 89.7% (35/39) and 70.6% (36/51) of all non-canonical domains respectively (Supplementary Table S3). These non-canonical domains may play a key role in the function of LRR-RLK genes. In the Arabidopsis phylogenetic tree, we show experimentally verified known receptor and co-receptor pairs and their interaction relationships (Figure 4C, Supplementary Table S4). Most of the receptors are distributed in evolutionary branches with ≥ 20 LRR units, except for AT5G62330 (ER) which has 19 LRR units. The co-receptor SERK family is distributed in the evolutionary branches with < 10 LRR units. Similarly, this phenomenon was also found in rice and tomato phylogenetic trees, except for Os01g0718300 (OsBRI1) which has 18 LRR units (Supplementary Figures S4B and S5B). In rice, four receptors interacting with two co-receptors are shown (Supplementary Table S5), including OsBRI1, XA21, XA3, OsFLS2, OsSERK1 and OsSERK2 [69,70,71,72]. In tomato, one receptor interacting with three co-receptors is shown (Supplementary Table S6), including SlFLS2, SlSERK1, SlSERK3A and SlSERK3B [73,74]. SlSERK1 is not successfully re-annotated by DeepLRR. We consider LRR-RLK genes with LRR units ≥20 to be potential receptors and LRR-RLK genes with LRR units <10 to be potential co-receptors.

## 4. Discussion

In the study, we introduce DeepLRR, an accurate website tool for the detection of LRR units from protein sequences and identification of NLR, RLK, RLP and its non-canonical domains. Compared to existing tools for LRR unit detection, DeepLRR attained a higher F1-score when using the set of 572 LRR proteins and maintained a great balance between precision and sensitivity. Analyzing the domain composition of LRR-RLK genes in Arabidopsis, rice and tomato, we found that LRR units and kinase domains of individual LRR-RLK genes overlap. In the future, we will try to improve the ability of DeepLRR to predict LRR units from protein sequences through a more comprehensive dataset, more structural information and usage of the capsule network [75]. In addition, we will also update the identification pipeline for more important proteins with the LRR domain and provide a more in-depth exploration of plant immune mechanisms based on DeepLRR. Through the phylogenetic trees of Arabidopsis, rice and tomato, we found that some LRR-RLK genes may be potential receptors or co-receptors.

## 5. Conclusions

DeepLRR combines deep learning algorithms and LRR features to predict the number and location of LRR units in an unknown protein sequence. By predicting the number of LRR units, it is possible to further study the interaction between receptors and co-receptors in plant immunity. In addition, DeepLRR can help researchers quickly identify disease-resistant proteins and their non-canonical domains in species of interest. This provides a reference for studying integrated decoy domains in plant immunity.

## Figures and Tables

**Table 1:** Performance of DeepLRR and six existing tools in predicting LRR units for 572 test protein sequences.

Method	Precision	Sensitivity	F1
LRRpredictor	0.582	**0.854**	0.692
LRRsearch	0.676	0.813	0.739
LRRfinder	0.798	0.669	0.728
Pfam	0.192	0.037	0.062
Prosite	**0.836**	0.379	0.522
Smart	0.398	0.167	0.235
DeepLRR	0.744	0.783	**0.763**

Prosite has the highest precision of 0.836 while LRRpredictor has the highest sensitivity of 0.854. The prediction result of DeepLRR achieves a great balance between precision and sensitivity (0.744, 0.783) and it has the highest F1-score of 0.763.

## Data Availability

All relevant codes and data of this research are uploaded to GitHub: (https://github.com/zhenyaliu77/DeepLRR; accessed on 28 November 2021).

## Supplementary Materials

The following supporting information can be downloaded at: https://www.mdpi.com/article/10.3390/plants11010136/s1, Figure S1. LRR positive sample dataset construction and analysis; Figure S2. LRR negative sample dataset construction and dataset division; Figure S3. Contour maps of DeepLRR prediction performance under different parameter combinations; Figure S4. Re-annotate the LRR-RLK gene in the rice genome, chromosome mapping, gene cluster analysis and phylogenetic analysis; Figure S5. Re-annotate the LRR-RLK gene in the tomato genome, chromosome mapping, gene cluster analysis and phylogenetic analysis; Table S1. LRR unit highly conserved segment pattern; Table S2. The performance of four different models on 5-fold cross-validation and test dataset; Table S3. Distribution of non-canonical domains of LRR-RLK genes in Arabidopsis, rice and tomato; Table S4. Experimentally validated receptor and co-receptor pairs in Arabidopsis; Table S5. Experimentally validated receptor and co-receptor pairs in rice; Table S6. Experimentally validated receptor and co-receptor pairs in tomato.
