# Supplementary material for: DeepLRR: An Online Webserver for Leucine-Rich-Repeat Containing Protein Characterization Based on Deep Learning
Source: Plants (Basel). 2022 Jan 4;11(1):136. doi: 10.3390/plants11010136 (PMC8796025; doi:10.3390/plants11010136)
Supplement: Supplementary file 1 [file plants-11-00136-s001.zip › plants-1508518-supplementary/Supplementary Figures-1.17.pdf]

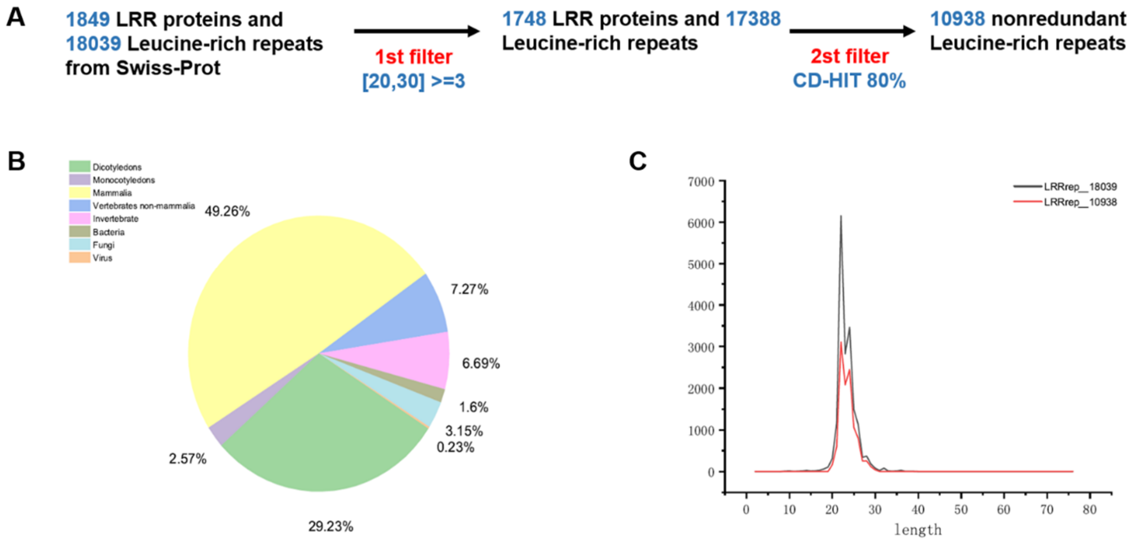

Figure S1. LRR positive sample dataset construction and analysis
(A) LRR positive sample dataset construction process. **(B)** Species distribution of 1748 LRR proteins. Mammalia (49.26%), Dicotyledon (29.23%), Vertebrate non-mammalia (7.27%), Invertebrate (6.69%), Fungi (3.15%), Monocotyledon (2.57%), Bacteria (1.6%) and Virus (0.23%). **(C)** Length distribution of 18039 LRR units and 10938 LRR units. For the 18039 LRR units in the original data, the proportion of the length between 20-30aa is 97.2%, the LRR unit with the largest number is 22aa, accounting for 34.2% and the LRR unit with least number is 30aa, accounting for 0.4%. For the 10938 LRR units after processing, the LRR unit with the largest number is 22aa, accounting for 28.4% and the LRR unit with the least number is 30aa, accounting for 0.35%.

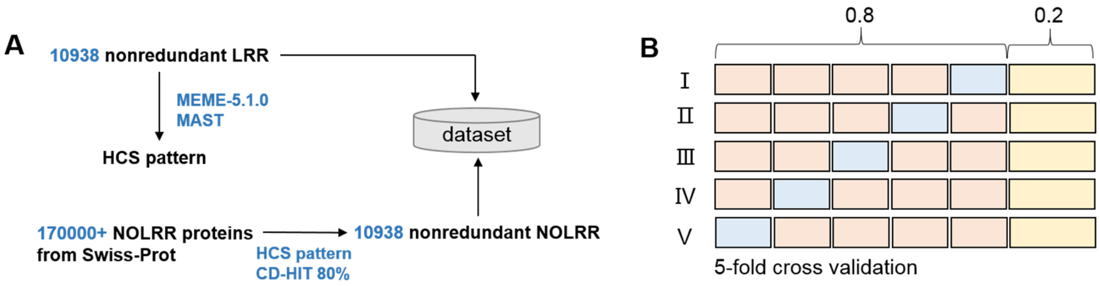

Figure S2. LRR negative sample dataset construction and dataset division
(A) LRR negative sample dataset construction process. **(B)**Training, validation, testing datasets construction process.

A

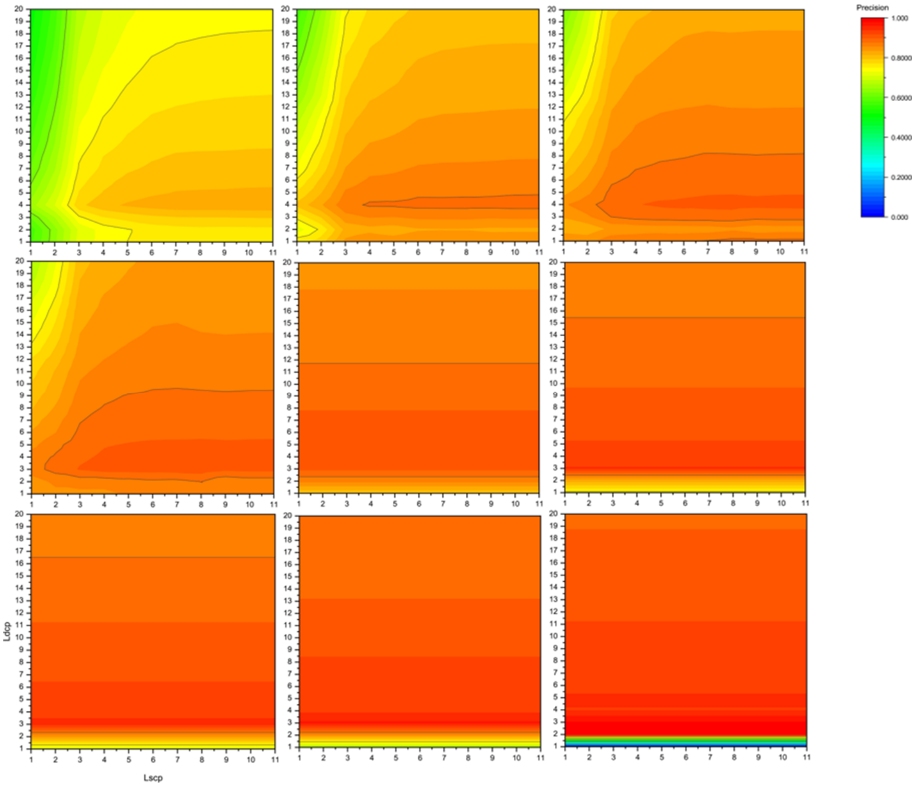
Precision
1.000
0.8000
0.6000
0.4000
0.2000
0.000
Ldcp
Lscp

B

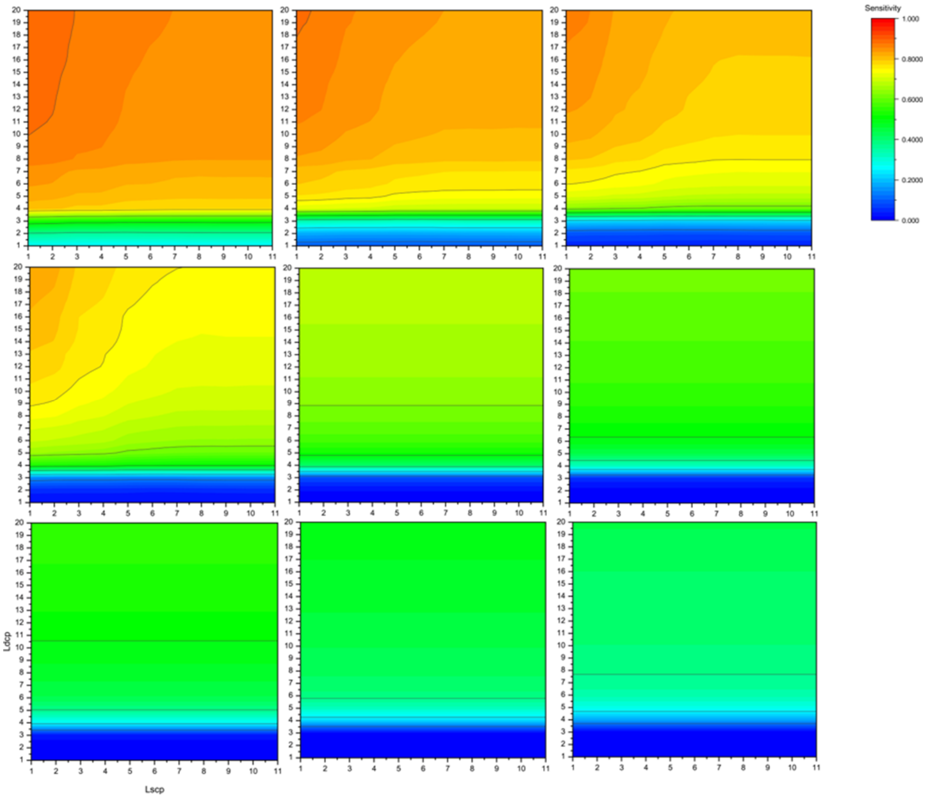
Sensitivity
1.000
0.8000
0.6000
0.4000
0.2000
0.000
Ldcp
Lscp

C

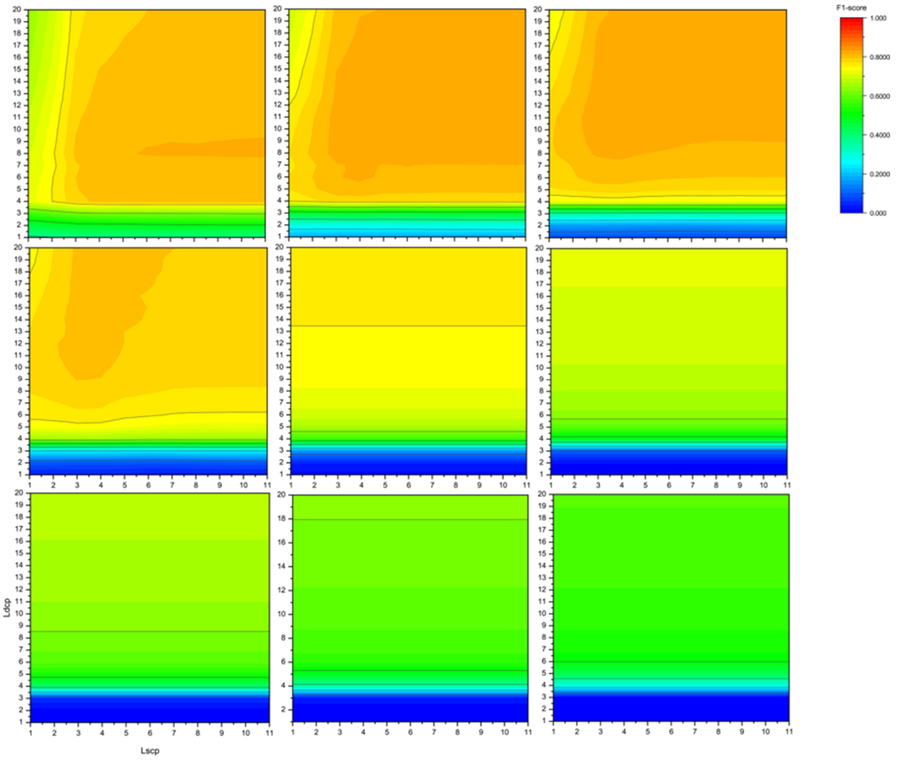

Figure S3. Contour maps of DeepLRR prediction performance under different parameter combinations

The x-axis of the small contour map represents the parameter Ldcp, which ranges from 1 to 20, and the y-axis represents the parameter Lscp, which ranges from 1 to 11. Each small contour map has the same parameter Lncp, which increases from left to right and from top to bottom, with a value range of 2 to 10. **(A)** Contour maps reflecting the precision of DeepLRR prediction results under different parameter combinations. **(B)** Contour maps reflecting the sensitivity of DeepLRR prediction results under different parameter combinations. **(C)** Contour maps reflecting the F1-score of DeepLRR prediction results under different parameter combine.

A

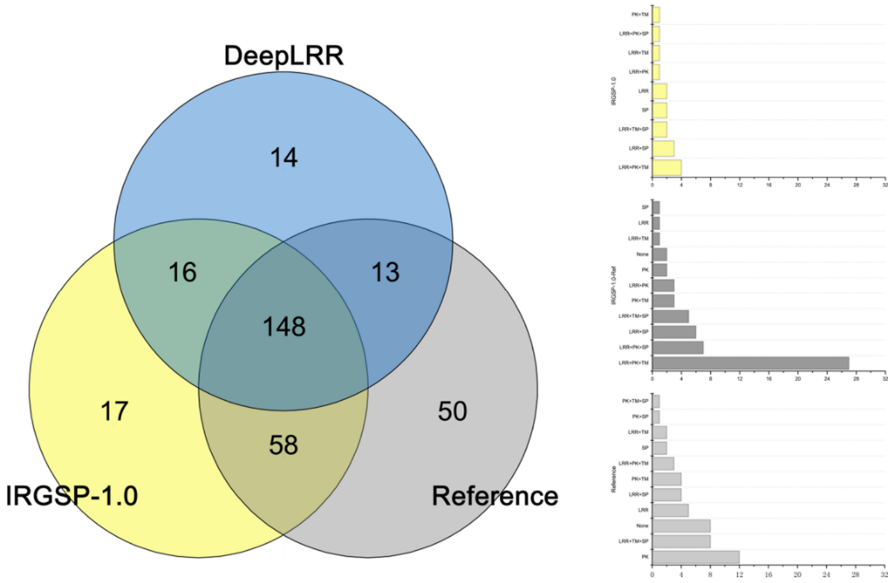
DeepLRR
14
16
13
148
17
58
50
IRGSP-1.0
Reference

B

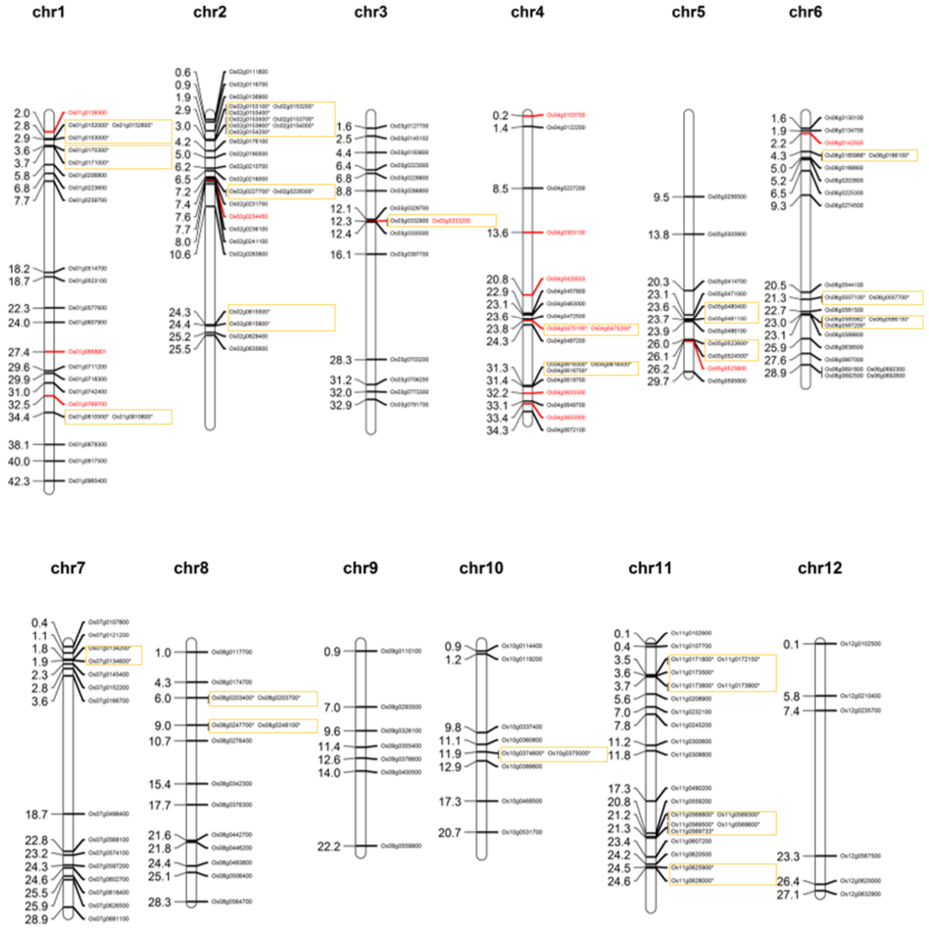
chr1
chr2
chr3
chr4
chr5
chr6
chr7
chr8
chr9
chr10
chr11
chr12

C

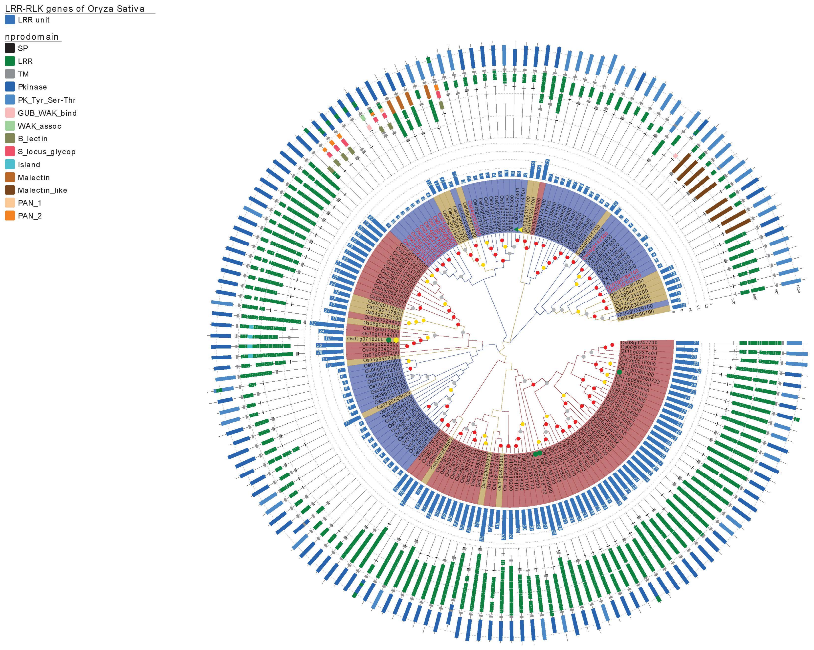

Figure S4. Re-annotate the LRR-RLK gene in the rice genome, chromosome mapping, gene cluster analysis and phylogenetic analysis

(A) The Venn diagram on the left shows DeepLRR, reference genome IRGSP-1.0 and representative paper respectively annotated results of the LRR-RLK gene in the rice genome. The histogram on the right shows the domain composition of the LRR-RLK gene that DeepLRR could not successfully annotate, including three datasets unique to IRGSP-1.0, shared by IRGSP-1.0 and representative paper and unique to representative paper. **(B)** Distribution of LRR-RLK genes were re-annotated by DeepLRR on the chromosomes of rice. The gold rectangles represent different gene clusters, tandem repeat genes are marked with an asterisk and gene names marked in red are LRR-RLK genes annotated only by DeepLRR. **(C)** An unrooted phylogenetic tree of LRR-RLK genes were re-annotated by DeepLRR in rice. The phylogenetic tree was established with amino acid sequences of the kinase domains by the neighbor-joining (NJ) method. The circles of different colors on the sub-nodes of the phylogenetic tree show different ranges of bootstrap values. The red circle shows the bootstrap value is from 0.9 to 1, the gold circle shows the bootstrap value is from 0.7 to 0.9 and the dark grey circle shows the bootstrap value is from 0.5 to 0.7. The different background colors of the leaf nodes indicate that the number of LRR units contained is in different ranges. Dark red indicates that the number of LRR units is greater than or equal to 20, dark yellow indicates that the number of LRR units is greater than or equal to 10 and less than 20, and dark blue indicates that the number of LRR units is less than 10. The histogram outside the leaf node shows the number of corresponding LRR units in detail. In addition, the phylogenetic tree shows the receptor and co-

receptor pairs that have been experimentally verified so far. The circle represents receptor, the triangle represents co-receptor, and the same color indicates that there is an interaction. Finally, domain composition of each LRR-RLK gene is shown in detail.

A

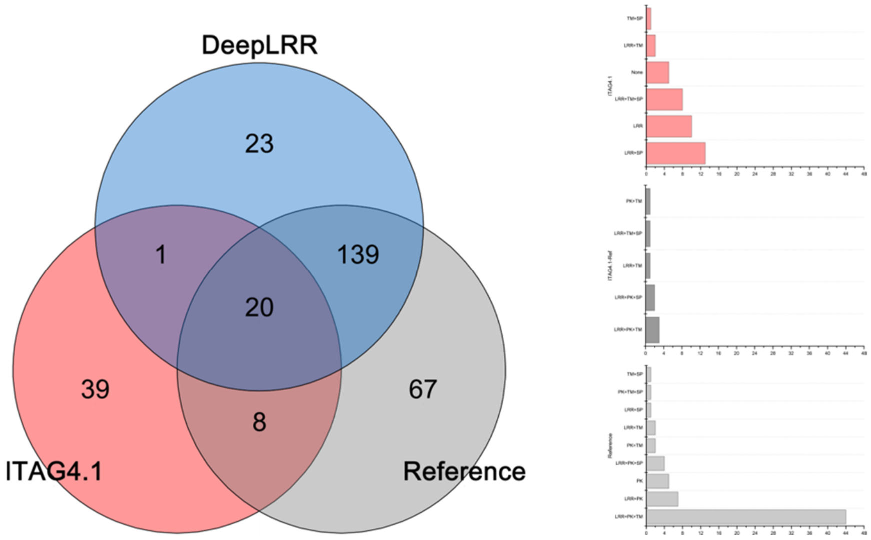
DeepLRR
23
1
139
20
39
67
8
ITAG4.1
Reference

B

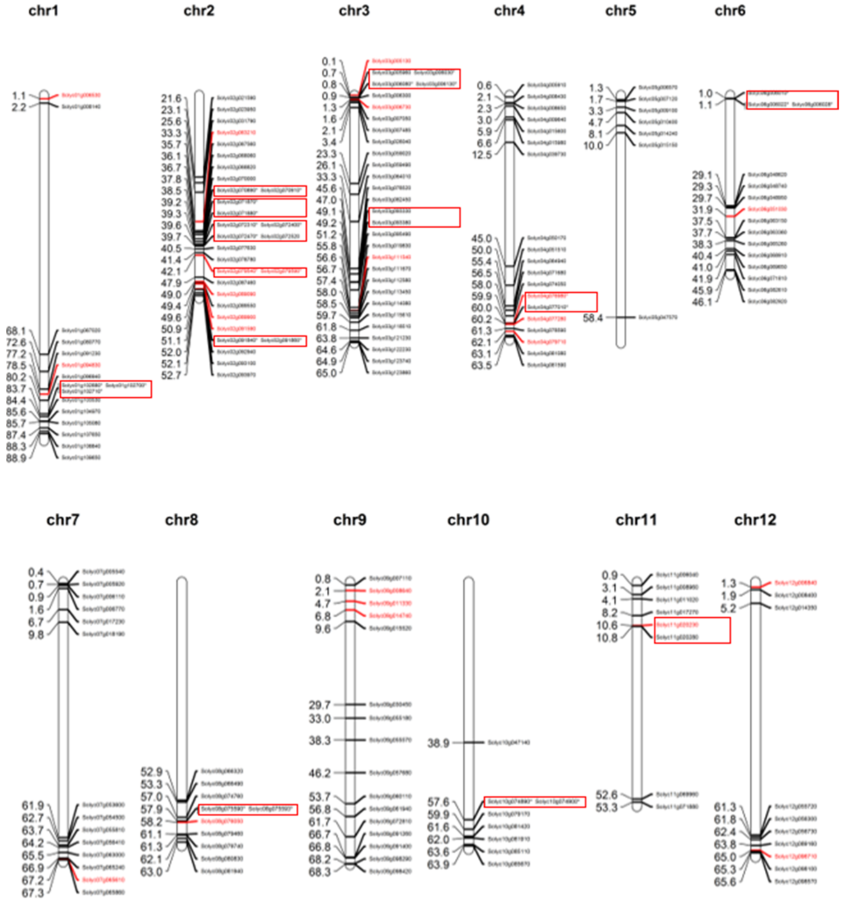
chr1
chr2
chr3
chr4
chr5
chr6
chr7
chr8
chr9
chr10
chr11
chr12

C

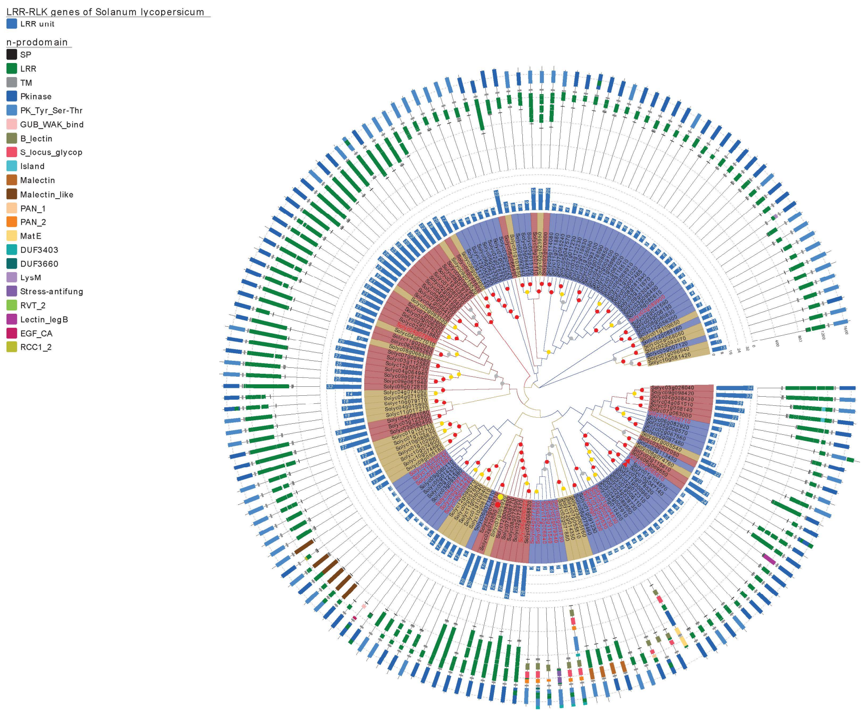

Figure S5. Re-annotate the LRR-RLK gene in the tomato genome, chromosome mapping, gene cluster analysis and phylogenetic analysis

(A) The Venn diagram on the left shows DeepLRR, reference genome ITAG4.1 and representative paper respectively annotated results of the LRR-RLK gene in the tomato genome. The histogram on the right shows the domain composition of the LRR-RLK gene that DeepLRR could not successfully annotate, including three datasets unique to ITAG4.1, shared by ITAG4.1 and representative paper and unique to representative paper. **(B)** Distribution of LRR-RLK genes were re-annotated by DeepLRR on the chromosomes of tomato. The red rectangles represent different gene clusters, tandem repeat genes are marked with an asterisk and gene names marked in red are LRR-RLK genes annotated only by DeepLRR. **(C)** An unrooted phylogenetic tree of LRR-RLK genes were re-annotated by DeepLRR in tomato. The phylogenetic tree was established with amino acid sequences of the kinase domains by the neighbor-joining (NJ) method. The circles of different colors on the sub-nodes of the phylogenetic tree show different ranges of bootstrap values. The red circle shows the bootstrap value is from 0.9 to 1, the gold circle shows the bootstrap value is from 0.7 to 0.9 and the dark grey circle shows the bootstrap value is from 0.5 to 0.7. The different background colors of the leaf nodes indicate that the number of LRR units contained is in different ranges. Dark red indicates that the number of LRR units is greater than or equal to 20, dark yellow indicates that the number of LRR units is greater than or equal to 10 and less than 20, and dark blue indicates that the number of LRR units is

less than 10. The histogram outside the leaf node shows the number of corresponding LRR units in detail. In addition, the phylogenetic tree shows the receptor and co-receptor pairs that have been experimentally verified so far. The circle represents receptor, the triangle represents co-receptor, and the same color indicates that there is an interaction. Finally, domain composition of each LRR-RLK gene is shown in detail.
