# Supplementary material for: DeepLRR: An Online Webserver for Leucine-Rich-Repeat Containing Protein Characterization Based on Deep Learning
Source: Plants (Basel). 2022 Jan 4;11(1):136. doi: 10.3390/plants11010136 (PMC8796025; doi:10.3390/plants11010136)
Supplement: Supplementary file 1 [file plants-11-00136-s001.zip › plants-1508518-supplementary/Supplementary Tables.pdf]

Table S1. LRR unit highly conserved segment pattern

**L**xxLxLxxNxL		Lxx**L**xLxxNxL		LxxLx**L**xxNxL		LxxLxLxx**N**xL		LxxLxLxxNx**L**	
**L**	0.8667	**L**	0.8311	**L**	0.7405	**N**	0.8899	**L**	0.4980
**I**	0.0516	**I**	0.0701	**V**	0.0845	**C**	0.0513	**I**	0.2585
**V**	0.0373	**V**	0.0491	**I**	0.0784	**T**	0.0256	**F**	0.1633
**M**	0.0144	**F**	0.0314	**F**	0.0346	**S**	0.0136	**V**	0.0242
**T**	0.0138	**M**	0.0146	**M**	0.0311	V	0.0028	**M**	0.0121
**F**	0.0052	A	0.0028	**A**	0.0230	A	0.0027	**S**	0.0073
A	0.0046	C	0.0004	**C**	0.0052	M	0.0021	**N**	0.0052
Y	0.0020	W	0.0001	T	0.0007	L	0.0020	K	0.0046
C	0.0011	T	0.0001	G	0.0007	Q	0.0016	R	0.0040
S	0.0007	S	0.0001	Y	0.0005	Y	0.0015	Q	0.0039
P	0.0007			W	0.0005	I	0.0012	E	0.0034
Q	0.0005			R	0.0001	G	0.0010	Y	0.0032
D	0.0005			Q	0.0001	K	0.0009	G	0.0024
N	0.0003					H	0.0008	D	0.0021
K	0.0003					D	0.0008	H	0.0017
G	0.0003					R	0.0007	A	0.0016
E	0.0001					P	0.0007	W	0.0015
						F	0.0005	T	0.0012
						W	0.0001	P	0.0011
								C	0.0008

The bolded amino acids in the first row represents highly conserved position in the HCSP, and the corresponding column shows the amino acids that appear in each conserved position and their frequency. Amino acids in bold are those with a frequency greater than or equal to 0.5%.

Table S2. The performance of four different models on 5-fold cross validation and test dataset

Classifiers	Cross-validation					Test
	CV1	CV2	CV3	CV4	CV5	
CNN	**0.9408**	**0.9416**	**0.9382**	**0.9316**	**0.9445**	**0.9405**
SVM	0.9202	0.9309	0.9208	0.9224	0.9294	0.9305
RF	0.8928	0.9089	0.8926	0.9014	0.9038	0.8953
NB	0.8554	0.8639	0.8376	0.8443	0.8568	0.8519

Classifiers	Cross-validation					Test
	CV1	CV2	CV3	CV4	CV5	
CNN	**0.8830**	**0.8848**	**0.8779**	**0.8661**	**0.8886**	**0.8831**
SVM	0.8398	0.8635	0.8421	0.8449	0.8599	0.8628
RF	0.7818	0.8168	0.7836	0.8000	0.8060	0.7893
NB	0.7078	0.7272	0.6722	0.6893	0.7134	0.7080

(Top) F1-score of CNN model and three machine learning models. **(Bottom)** MCC of CNN model and three machine learning models.

Table S3. Distribution of non-canonical domains of LRR-RLK genes in Arabidopsis, rice and tomato

	Arabidopsis	Rice	Tomato
B_lectin	2	7	10
S_locus_glycop	1	6	7
Malectin_like	43	9	6
PAN_2	2	5	6
Malectin	11	4	5
DUF3403	-		5
Island	4	4	2
GUB_WAK_bind	-	2	1
PAN_1	-	1	1
MatE	-	-	1
DUF3660	-	-	1
LysM	-	-	1
Stress-antifung	4	-	1
RVT_2	-	-	1
Lectin_legB	6	-	1
EGF_CA	-	-	1
RCC1_2	1	-	1
Ribonuc_2-5A	1	-	-
WAK_assoc	-	1	-

This table shows how many LRR-RLK genes in Arabidopsis, rice and tomato contain specific types of non-canonical domains. For example, there are two LRR-RLK genes in Arabidopsis that contain the B_lectin domain.

Table S4. Experimentally validated receptor and co-receptor pairs in Arabidopsis

	AT1G71828 (SERK1)	AT1G34210 (SERK2)	AT4G33430 (SERK3)	AT2G13790 (SERK4)	AT2G13800 (SERK5)
AT5G46330(FLS2)	-	-	+	+	-
AT5G20480(EFR)	-	-	+	+	-
AT1G73080(PEPR1)	-	-	+	+	-
AT1G17750(PEPR2)	-	-	+	+	-
AT4G39400(BRI1)	+	-	+	+	-
AT5G07280(EMS1)	+	+	-	-	-
AT5G65710(HSL2)	+	+	+	+	-
AT4G28490(HAE)	+	+	+	+	-
AT2G02220(PSKR1)	+	+	+	-	-
AT2G26330(ER)	+	+	+	+	-
AT5G62230(ERL1)	+	+	+	+	-

This table shows the experimentally verified receptor and co-receptor pairs in Arabidopsis and whether there is an interaction between them. The first column is receptor gene, the first row is co-receptor gene, and the plus sign indicates that there is an interaction between receptor and co-receptor.

Table S5. Experimentally validated receptor and co-receptor pairs in rice

	Os28g0174700(OsSERK1)	Os04g0457800(OsSERK2)
Os01g0718300(OsBRI1)	+	+
Os11g0569733(XA21)	-	+
Os11g0208900(XA3)	-	+
Os04g0618700(OsFLS2)	-	+

This table shows the experimentally verified receptor and co-receptor pairs in rice and whether there is an interaction between them. The first column is receptor gene, the first row is co-receptor gene, and the plus sign indicates that there is an interaction between receptor and co-receptor.

Table S6. Experimentally validated receptor and co-receptor pairs in tomato

	Solyc04g072570 (SlSERK1)	Solyc10g047140 (SlSERK3A)	Solyc01g104970 (SlSERK3B)
Solyc02g070890(SlFLS2)	+	+	+

This table shows the experimentally verified receptor and co-receptor pairs in tomato and whether there is an interaction between them. The first column is receptor gene, the first row is co-receptor gene, and the plus sign indicates that there is an interaction between receptor and co-receptor.
